# Jensen–Steffensen inequality for strongly convex functions

**DOI:** 10.1186/s13660-018-1897-2

**Published:** 2018-11-12

**Authors:** M. Klaričić Bakula

**Affiliations:** Faculty of Science, University of Split, Split, Croatia

**Keywords:** 26D15, 39B62, 26B25, Strongly convex functions, Jensen inequality, Jensen–Steffensen inequality

## Abstract

The Jensen inequality for convex functions holds under the assumption that all of the included weights are nonnegative. If we allow some of the weights to be negative, such an inequality is called the Jensen–Steffensen inequality for convex functions. In this paper we prove the Jensen–Steffensen inequality for strongly convex functions.

## Introduction

Let $I\subset{\mathbb{R}}$ be an interval. It is well known that if a function $f:I\rightarrow{\mathbb{R}}$ is convex, then

1.1 $$ f \Biggl( \sum_{i=1}^{n}p_{i}x_{i} \Biggr) \leq\sum_{i=1}^{n}p_{i}f(x_{i}) $$

for all $n\in{\mathbb{N}}$, $x_{1},\ldots,x_{n}\in I$, and $p_{1},\ldots,p_{n}>0$ with $p_{1}+\cdots+p_{n}=1$. If *f* is strictly convex, then (1.1) is strict unless all $x_{i}$ are equal [7, p. 43]. This classical *Jensen inequality* is one of the most important inequalities in convex analysis, and it has various applications in mathematics, statistics, economics, and engineering sciences.

It is also known that the assumption $p_{1},\ldots,p_{n}>0$ can be relaxed at the expense of restricting $x_{1},\ldots,x_{n}$ more severely [9]. Namely, if $\boldsymbol{p}= ( p_{1},\ldots,p_{n} ) $ is a real *n*-tuple such that for every $k\in \{ 1,\ldots,n \} $

1.2 $$ 0\leq p_{1}+\cdots+p_{k}\leq p_{1}+ \cdots+p_{n}=1, $$

then for any monotonic *n*-tuple $\boldsymbol{x}= ( x_{1},\ldots ,x_{n} ) \in I^{n}$ (increasing or decreasing) we get

$$\overline{x}=p_{1}x_{1}+\cdots+p_{n}x_{n} \in I, $$

and for any function *f* convex on *I* (1.1) still holds. Under such assumptions (1.1) is called *the Jensen–Steffensen inequality* for convex functions and (1.2) are called *Steffensen’s conditions* due to J. F. Steffensen. Again, for a strictly convex function *f*, (1.1) remains strict under certain additional assumptions on ***x*** and ***p*** [1]. It is needless to say that a mathematical mind has to question the limitation $p_{1},\ldots,p_{n}>0$ even if in the usual practice we can cope with it.

Variants of the Jensen inequality are proved for various classes of generalized convex functions, and the class of strongly convex functions is among them. Recall that a function $f:I\rightarrow{\mathbb{R}}$ is called *strongly convex with modulus* $c>0$ if

1.3 $$ f\bigl(tx+(1-t)y\bigr)\leq tf(x)+(1-t)f(y)-ct(1-t) (x-y)^{2} $$

for all $x,y\in I$ and $t\in{}[0,1]$ [8]. Strongly convex functions are useful in optimization theory, mathematical economics and approximation theory, and an interested reader can find more about them in an excellent survey paper [5].

As we can easily see, strong convexity is a strengthening of the notion of convexity, and some properties of strongly convex functions are just “stronger versions” of analogous properties of convex functions (for more details, see [5]). One example of such a stronger version is the Jensen inequality for strongly convex functions (see [4] or [5]). If $f:I\rightarrow{\mathbb{R}}$, $I\subset{\mathbb{R}}$, is strongly convex with modulus *c*, then

1.4 $$ f\Biggl(\sum_{i=1}^{n}p_{i}x_{i} \Biggr)\leq\sum_{i=1}^{n}p_{i}f(x_{i})-c \sum_{i=1}^{n}p_{i}(x_{i}- \bar{x})^{2} $$

for all $x_{1},\ldots,x_{n}\in I$ and all $p_{1},\ldots,p_{n}>0$ such that $p_{1}+\cdots+p_{n}=1$. If we compare (1.4) with (1.1), we see that (1.4) provides a better upper bound for $f(\bar{x})$ since the term $c\sum_{i=1}^{n}p_{i}(x_{i}-\bar{x})^{2}$ is always nonnegative. Of course, if $c=0$, we go right back to convex functions and (1.1).

We must emphasize here that proving a Jensen type inequality for some class of generalized convex functions does not necessarily mean that such inequality holds under Steffensen’s conditions. The goal of this paper is to prove that for the class of strongly convex functions this is not the case.

## Main result

Strongly convex functions have a very useful characterization: they always have a specific convex representation. This is stated in the following theorem (see [3] or [6]).

### Theorem 1

*Let* *I* *be an interval in* ${\mathbb{R}}$. *A function* $f:I\rightarrow{\mathbb{R}}$ *is strongly convex with modulus* *c* *if and only if the function* $g=f-c ( \cdot ) ^{2}$ *is convex*.

The Jensen inequality for strongly convex functions can be proved either using Theorem 1 and the Jensen inequality for convex functions or (for *I* open) directly, using the “support parabola” property [5, Theorem 1]. In this section we prove the Jensen–Steffensen inequality for strongly convex functions using Theorem 1.

In the rest of the paper we use the following notation related to the *n*-tuples $\boldsymbol{x}= ( x_{1},\ldots,x_{n} ) $ and $\boldsymbol{p}= ( p_{1},\ldots,p_{n} ), n\in \mathbb{N} $:

$$\begin{aligned} & \bar{x} =p_{1}x_{1}+\cdots+p_{n}x_{n}, \\ &P_{k} =p_{1}+\cdots+p_{k}, \quad k\in \{ 1,2, \ldots,n \}, \\ & \overline{P}_{k} =p_{k}+\cdots+p_{n},\quad k\in \{ 1,2,\ldots ,n \}. \end{aligned}$$

### Theorem 2

*Let* *I* *be an interval in* ${\mathbb{R}}$. *If* $f:I\rightarrow {\mathbb{R}}$ *is a strongly convex function with modulus* *c*, *then for every monotonic* *n*-*tuple* $\boldsymbol{x}= ( x_{1},\ldots,x_{n} ) \in I^{n}$ *and every real* *n*-*tuple* $\boldsymbol{p}= ( p_{1},\ldots ,p_{n} ) $ *such that*, *for every* $i\in \{ 1,2,\ldots ,n \}$,

$$0\leq P_{i}\leq P_{n}=1 $$

*the following inequality holds*:

$$f \Biggl( \sum_{i=1}^{n}p_{i}x_{i} \Biggr) \leq\sum_{i=1}^{n}p_{i}f(x_{i})-c \sum_{i=1}^{n}p_{i}(x_{i}- \bar{x})^{2}. $$

### Proof

Suppose that ***x*** is increasing (for ***x*** decreasing the proof is analogous). It can be easily seen that Steffensen’s conditions yield

$$\overline{P}_{k}\geq0,\quad k\in \{ 1,2,\ldots,n \}, $$

and

$$x_{n}-\bar{x}=P_{n} ( x_{n}-\bar{x} ) =\sum _{i=1}^{n-1}P_{i}(x_{i+1}-x_{i}) \geq0, $$

hence we obtain $\bar{x}\leq x_{n}$. Analogously,

$$\bar{x}-x_{1}=P_{n} ( \bar{x}-x_{1} ) =\sum _{i=2}^{n}\overline {P}_{i}(x_{i}-x_{i-1}) \geq0, $$

and $x_{1}\leq\bar{x}$. From that we may conclude $\bar{x}\in [ x_{1},x_{n} ] \subset I$, which means that $g ( \bar {x} ) =g ( \sum_{i=1}^{n}p_{i}x_{i} ) $ is defined.

Using the convex representation $g=f-c ( \cdot ) ^{2}$ as in Theorem 1 and applying the Jensen–Steffensen inequality for convex functions, we obtain

$$g\Biggl(\sum_{i=1}^{n}p_{i}x_{i} \Biggr)\leq\sum_{i=1}^{n}p_{i}g(x_{i}). $$

Returning back to *f*, we get

$$\begin{aligned} f \Biggl( \sum_{i=1}^{n}p_{i}x_{i} \Biggr) -c \Biggl( \sum_{i=1}^{n}p_{i}x_{i} \Biggr) ^{2} & \leq\sum_{i=1}^{n}p_{i} \bigl( f(x_{i})-cx_{i}^{2} \bigr) \\ & =\sum_{i=1}^{n}p_{i}f(x_{i})-c \sum_{i=1}^{n}p_{i}x_{i}^{2}, \end{aligned}$$

or written differently

$$\begin{aligned} f \Biggl( \sum_{i=1}^{n}p_{i}x_{i} \Biggr) & \leq\sum_{i=1}^{n}p_{i}f(x_{i})-c \Biggl[ \sum_{i=1}^{n}p_{i}x_{i}^{2}- \Biggl( \sum_{i=1}^{n}p_{i}x_{i} \Biggr) ^{2} \Biggr] \\ & =\sum_{i=1}^{n}p_{i}f(x_{i})-c \Biggl[ \sum_{i=1}^{n}p_{i}x_{i}^{2}- \bar {x}^{2} \Biggr] \\ & =\sum_{i=1}^{n}p_{i}f(x_{i})-c \Biggl[ \sum_{i=1}^{n}p_{i}x_{i}^{2}-2 \bar {x}^{2}+\bar{x}^{2} \Biggr] \\ & =\sum_{i=1}^{n}p_{i}f(x_{i})-c \Biggl[ \sum_{i=1}^{n}p_{i}x_{i}^{2}-2 \bar {x}\sum_{i=1}^{n}p_{i}x_{i}+ \bar{x}^{2}\sum_{i=1}^{n}p_{i} \Biggr] \\ & =\sum_{i=1}^{n}p_{i}f(x_{i})-c \sum_{i=1}^{n}p_{i}(x_{i}- \bar{x})^{2}. \end{aligned}$$

 □

## Alternative reproach

What would happen if we try to prove (1.4) under Steffensen’s conditions directly using the support parabola property? The question is not without sense since in the case of the Jensen inequality for strongly convex functions both ways produce the same inequality as in (1.4) but, generally speaking, any negative weights in ***p*** can at some place interrupt the chain of conclusions in a proof. This is exactly the reason why it is considerably more difficult to prove (1.1) under Steffensen’s conditions. We will see what happens in this case in the next theorem, but first we need the following lemma which basically says that the support parabola in $x_{0}$ can be “shifted up” from $x_{0}$ to *y* and still remain “under” $f ( x ) $ if $x\leq y\leq x_{0}$.

### Lemma 1

*Let* $I\subset{\mathbb{R}}$ *be an open interval*, *let* $f:I\rightarrow {\mathbb{R}}$ *be a strongly convex function with modulus* *c*, *and for* $x_{0}\in I$ *let*

3.1 $$ y=f ( x_{0} ) +\lambda ( x-x_{0} ) +c(x-x_{0})^{2} $$

*be the support parabola for* *f* *in* $x_{0}$. *Then for every* $x,y\in I$ *such that* $x\leq y\leq x_{0}$

3.2 $$ f ( x ) -f ( y ) \geq\lambda ( x-y ) +c(x-y)^{2}, $$

*and for* $x,y\in I$ *such that* $x_{0}\leq x\leq y$

3.3 $$ f ( y ) -f ( x ) \geq\lambda ( y-x ) +c(y-x)^{2}. $$

### Proof

Since (3.1) is a support parabola for *f* in $x_{0}$, it follows that for every $x\in I$

3.4 $$ f(x)-f ( x_{0} ) \geq\lambda ( x-x_{0} ) +c(x-x_{0})^{2}. $$

Let $x,y\in I$ be such that $x< y< x_{0}$. The middle element *y* can be represented as a convex combination of *x* and *z* in the following way:

$$y=\frac{x_{0}-y}{x_{0}-x}x+\frac{y-x}{x_{0}-x}x_{0}. $$

From the definition of strong convexity we have

$$f ( y ) \leq\frac{x_{0}-y}{x_{0}-x}f ( x ) +\frac {y-x}{x_{0}-x}f ( x_{0} ) -c \frac{x_{0}-y}{x_{0}-x}\frac {y-x}{x_{0}-x}(x-x_{0})^{2}, $$

and since

$$\frac{x_{0}-y}{x_{0}-x}+\frac{y-x}{x_{0}-x}=1, $$

we can write

$$\begin{aligned} f ( y ) &=\frac{x_{0}-y}{x_{0}-x}f ( y ) +\frac {y-x}{x_{0}-x}f ( y ) \\ & \leq\frac{x_{0}-y}{x_{0}-x}f ( x ) +\frac {y-x}{x_{0}-x}f ( x_{0} ) -c \frac{x_{0}-y}{x_{0}-x}\frac{y-x}{x_{0}-x}(x-x_{0})^{2}. \end{aligned}$$

After a simple calculation we obtain

$$( x_{0}-y ) \bigl( f ( x ) -f ( y ) \bigr) \geq ( x-y ) \bigl( f ( x_{0} ) -f ( y ) \bigr) +c\frac{ ( x_{0}-y ) ( y-x ) }{x_{0}-x}(x-x_{0})^{2} $$

and

3.5 $$ \frac{f ( x ) -f ( y ) }{x-y}\leq\frac{f ( x_{0} ) -f ( y ) }{x_{0}-y}-c(x_{0}-x). $$

The support parabola property (3.4) gives

$$f(y)-f ( x_{0} ) \geq\lambda ( y-x_{0} ) +c(y-x_{0})^{2}, $$

and since $y-x_{0}<0$

$$\frac{f(x_{0})-f ( y ) }{x_{0}-y}\leq\lambda-c(x_{0}-y). $$

Using the above inequality and (3.5), we obtain

$$\begin{aligned} \frac{f ( x ) -f ( y ) }{x-y} & \leq\frac {f ( x_{0} ) -f ( y ) }{x_{0}-y}-c(x_{0}-x) \\ & \leq\lambda-c(x_{0}-y)-c(x_{0}-x)=\lambda+c ( x+y-2x_{0} ). \end{aligned}$$

Since $x-y<0$ we get

$$f ( x ) -f ( y ) \geq\lambda ( x-y ) +c(x-y) ( x+y-2x_{0} ), $$

and because of $x+y-2x_{0}< x-y$, we end up with

$$f ( x ) -f ( y ) \geq\lambda ( x-y ) +c(x-y)^{2}. $$

If $x_{0}< x< y$, in an analogous way we can prove

$$f ( y ) -f ( x ) \geq\lambda ( y-x ) +c(y-x)^{2}. $$

Note that the above inequalities still hold in the trivial way if $x=y$. □

### Remark 1

(3.2) and (3.3) can be also proved using the convex representation $g=f-c ( \cdot ) ^{2}$. We start from the support parabola property in $x_{0}\in I$

$$f(x)-f ( x_{0} ) \geq\lambda ( x-x_{0} ) +c(x-x_{0})^{2}. $$

Then

$$g(x)-g ( x_{0} ) +cx^{2}-cx_{0}^{2} \geq\lambda ( x-x_{0} ) +c(x-x_{0})^{2}, $$

that is,

$$\begin{aligned} g(x)-g ( x_{0} ) & \geq\lambda ( x-x_{0} ) +c(x-x_{0})^{2}-cx^{2}+cx_{0}^{2} \\ & = ( \lambda-2cx_{0} ) ( x-x_{0} ) =\lambda ^{\prime } ( x-x_{0} ), \end{aligned}$$

hence *g* has a support line in $x_{0}$ for $\lambda^{\prime}=\lambda -2cx_{0}$. Since *g* is convex, we know that for every $x_{0}\leq x\leq y$ [7]

$$g(y)-g ( x ) \geq\lambda^{\prime} ( y-x ) = ( \lambda-2cx_{0} ) ( y-x ). $$

Returning to *f*, we obtain

$$f ( y ) -cy^{2}-f ( x ) +cx^{2}\geq ( \lambda-2cx_{0} ) ( y-x ), $$

hence

$$\begin{aligned} f ( y ) -f ( x ) & \geq ( \lambda -2cx_{0} ) ( y-x ) +cy^{2}-cx^{2} \\ & =\lambda ( y-x ) +c ( y-x ) ( x+y-2x_{0} ) \\ & \geq\lambda ( y-x ) +c ( y-x ) ( x+y-2x ) \\ & =\lambda ( y-x ) +c ( y-x ) ^{2}. \end{aligned}$$

Consequently,

$$f ( y ) -f ( x ) \geq\lambda ( y-x ) +c(y-x)^{2}, \quad x_{0}\leq x\leq y. $$

Analogously, we can prove

$$f ( x ) -f ( y ) \geq\lambda ( x-y ) +c(x-y)^{2},\quad x\leq y\leq x_{0}. $$

### Theorem 3

*Let* $I\subset{\mathbb{R}}$ *be an open interval*. *If* $f:I\rightarrow {\mathbb{R}}$ *is a strongly convex function with modulus* *c*, *then for every monotonic* *n*-*tuple* $\boldsymbol{x}= ( x_{1},\ldots,x_{n} ) \in I^{n}$ *and every real* *n*-*tuple* $\boldsymbol{p}= ( p_{1},\ldots ,p_{n} ) $ *such that for every* $i\in \{ 1,2,\ldots,n \} $

$$0\leq P_{i}\leq P_{n}=1, $$

*there exists* $k\in \{ 1,\ldots,n-1 \} $ *such that* $\bar{x} \in [ x_{k},x_{k+1} ] $ *for* ***x*** *increasing or* $\bar {x}\in [ x_{k+1},x_{k} ] $ *for* ***x*** *decreasing*, *and*

$$\begin{aligned} & \sum_{i=1}^{n}p_{i}f(x_{i})-f \Biggl( \sum_{i=1}^{n}p_{i}x_{i} \Biggr) \\ &\quad \geq c \Biggl[ \sum_{i=1}^{k-1}P_{i}(x_{i}-x_{i+1})^{2}+P_{k}(x_{k}- \bar {x})^{2}+\overline{P}_{k+1}(x_{k+1}- \bar{x})^{2}+\sum_{i=k+2}^{n} \overline {P}_{i}(x_{i}-x_{i-1})^{2} \Biggr] \\ &\quad \geq0. \end{aligned}$$

### Proof

Suppose that ***x*** is increasing (for ***x*** decreasing the proof is analogous).

First observe that as in Theorem 2 we know that $\bar {x}\in [ x_{1},x_{n} ] \subset I$, and we may conclude that there exists some $k\in \{ 1,\ldots,n-1 \} $ such that $\bar{x}\in [ x_{k},x_{k+1} ]$.

From (3.4), choosing $x_{0}=\bar{x}$, we get

$$f(x)-f ( \bar{x} ) \geq\lambda ( x-\bar{x} ) +c(x-\bar{x})^{2}$$

for some $\lambda\in \mathbb{R} $ and every $x\in I$.

Next we use the Abel transformation to obtain the identities (similar can be found in [1])

3.6 $$\begin{aligned} 0={}&\sum_{i=1}^{n}p_{i}x_{i}- \bar{x} \\ ={}&\sum_{i=1}^{k-1}P_{i} ( x_{i}-x_{i+1} ) +P_{k} ( x_{k}- \bar{x} ) \\ &{} +\overline{P}_{k+1} ( x_{k+1}-\bar{x} ) +\sum _{i=k+2}^{n}\overline{P}_{i} ( x_{i}-x_{i-1} ) \end{aligned}$$

and

3.7 $$\begin{aligned} & \sum_{i=1}^{n}p_{i}f(x_{i})-f ( \bar{x} ) \\ &\quad =\sum_{i=1}^{k-1}P_{i} \bigl( f ( x_{i} ) -f ( x_{i+1} ) \bigr) +P_{k} \bigl( f ( x_{k} ) -f ( \bar{x} ) \bigr) \\ &\qquad{} +\overline{P}_{k+1} \bigl( f ( x_{k+1} ) -f ( \bar {x} ) \bigr) +\sum_{i=k+2}^{n}\overline{P}_{i} \bigl( f ( x_{i} ) -f ( x_{i-1} ) \bigr), \end{aligned}$$

where in the case $k=1$ we assume $\sum_{i=1}^{k-1}$ to be 0, while in the case $k=n-1$ we assume $\sum_{i=k+2}^{n}$ to be 0.

From (3.7), using (3.2), (3.3), and then (3.6), we get

$$\begin{aligned} & \sum_{i=1}^{n}p_{i}f(x_{i})-f ( \bar{x} ) \\ & \quad\geq\sum_{i=1}^{k-1}P_{i} \bigl( \lambda ( x_{i}-x_{i+1} ) +c(x_{i}-x_{i+1})^{2} \bigr) +P_{k} \bigl( \lambda ( x_{k}-\bar {x} ) +c(x_{k}-\bar{x})^{2} \bigr) \\ &\qquad{} +\overline{P}_{k+1} \bigl( \lambda ( x_{k+1}-\bar{x} ) +c(x_{k+1}-\bar{x})^{2} \bigr) +\sum _{i=k+2}^{n}\overline {P}_{i} \bigl( \lambda ( x_{i}-x_{i-1} ) +c(x_{i}-x_{i-1})^{2} \bigr) \\ & \quad =\lambda \Biggl[ \sum_{i=1}^{k-1}P_{i} ( x_{i}-x_{i+1} ) +P_{k} ( x_{k}- \bar{x} ) +\overline{P}_{k+1} ( x_{k+1}-\bar {x} ) +\sum _{i=k+2}^{n}\overline{P}_{i} ( x_{i}-x_{i-1} ) \Biggr] \\ &\qquad{} +c \Biggl[ \sum_{i=1}^{k-1}P_{i}(x_{i}-x_{i+1})^{2}+P_{k}(x_{k}- \bar {x})^{2}+\overline{P}_{k+1}(x_{k+1}- \bar{x})^{2}+\sum_{i=k+2}^{n} \overline{P}_{i}(x_{i}-x_{i-1})^{2} \Biggr] \\ &\quad =c \Biggl[ \sum_{i=1}^{k-1}P_{i}(x_{i}-x_{i+1})^{2}+P_{k}(x_{k}- \bar {x})^{2}+\overline{P}_{k+1}(x_{k+1}- \bar{x})^{2}+\sum_{i=k+2}^{n} \overline{P}_{i}(x_{i}-x_{i-1})^{2} \Biggr]. \end{aligned}$$

 □

It was hopeful to think that this way we can end up with

$$\sum_{i=1}^{n}p_{i}f(x_{i})-f ( \bar{x} ) \geq c\sum_{i=1}^{n}p_{i}(x_{i}- \bar{x})^{2}$$

since this is exactly what happens in the analogous proofs (direct and indirect) for convex functions. It would be possible if

3.8 $$\begin{aligned} & \sum_{i=1}^{k-1}P_{i}(x_{i}-x_{i+1})^{2}+P_{k}(x_{k}- \bar{x})^{2}+\overline{P}_{k+1}(x_{k+1}- \bar{x})^{2}+\sum_{i=k+2}^{n} \overline {P}_{i}(x_{i}-x_{i-1})^{2} \\ & \quad \geq\sum_{i=1}^{n}p_{i}(x_{i}- \bar{x})^{2}, \end{aligned}$$

but sadly this is not generally true.

### Example 1

Let $\boldsymbol{x}= ( 1,2,3,4 ) $, $\boldsymbol{p}= ( 1,-1,0,1 )$. Then

$$\begin{aligned} & P_{1} =1,\qquad P_{2}=0,\qquad P_{3}=0,\qquad P_{4}=1, \\ &\overline{P}_{1} =1, \qquad \overline{P}_{2}=0,\qquad \overline{P}_{3}=1, \qquad \overline {P}_{4}=1, \\ &\bar{x}=3\in [ 2,3 ],\quad k=2\ (\text{or } k=3), \\ & \sum_{i=1}^{1}P_{i}(x_{i}-x_{i+1})^{2}+P_{2}(x_{2}- \bar{x})^{2}+\overline{P}_{3}(x_{3}- \bar{x})^{2}+\sum_{i=4}^{4} \overline{P}_{i}(x_{i}-x_{i-1})^{2} \\ & \quad = ( 1-2 ) ^{2}+0+ ( 3-3 ) ^{2}+ ( 4-3 ) ^{2}=2, \\ &\sum_{i=1}^{4}p_{i}(x_{i}- \bar{x})^{2}= ( 1-3 ) ^{2}- ( 2-3 ) ^{2}+0+ ( 4-3 ) ^{2}=4>2. \end{aligned}$$

In fact, the following theorem holds.

### Theorem 4

*Let* $f, \boldsymbol{p}, \boldsymbol{x}$, *and* *k* *be as in Theorem *3. *Then*

$$\begin{aligned} & \sum_{i=1}^{k-1}P_{i}(x_{i}-x_{i+1})^{2}+P_{k}(x_{k}- \bar{x})^{2}+\overline{P}_{k+1}(x_{k+1}- \bar{x})^{2}+\sum_{i=k+2}^{n} \overline {P}_{i}(x_{i}-x_{i-1})^{2} \\ &\quad \leq\sum_{i=1}^{n}p_{i}(x_{i}- \bar{x})^{2}. \end{aligned}$$

### Proof

For the sake of simplicity, we introduce the following notation:

$$\begin{aligned} &I_{k} =\sum_{i=1}^{k-1}P_{i}(x_{i}-x_{i+1})^{2}+P_{k}(x_{k}- \bar{x})^{2}+\overline{P}_{k+1}(x_{k+1}- \bar{x})^{2}+\sum_{i=k+2}^{n} \overline{P}_{i}(x_{i}-x_{i-1})^{2}, \\ &\overline{\boldsymbol{x}^{2}} =\sum_{i=1}^{n}p_{i}x_{i}^{2}. \end{aligned}$$

Suppose that ***x*** is increasing (for ***x*** decreasing the proof is analogous). First note that for *k* as in Theorem 3 we have

$$\begin{aligned} &x_{i} \leq\bar{x},\quad i=1,2,\ldots,k, \\ &\bar{x} \leq x_{i},\quad i=k+1,\ldots,n. \end{aligned}$$

Using this notation, we get

$$\begin{aligned} I_{k}={}& \sum_{i=1}^{k-1}P_{i} \bigl( x_{i}^{2}-x_{i+1}^{2} \bigr) +P_{k}\bigl(x_{k}^{2}-\overline{ \boldsymbol{x}^{2}}\bigr)+\overline {P}_{k+1} \bigl(x_{k+1}^{2}-\overline{\boldsymbol{x}^{2}} \bigr)+\sum_{i=k+2}^{n}\overline {P}_{i} \bigl( x_{i}^{2}-x_{i-1}^{2} \bigr) \\ &{} -2\sum_{i=1}^{k-1}P_{i}x_{i}x_{i+1}+2 \sum_{i=1}^{k-1}P_{i}x_{i+1}^{2}+P_{k} \bigl(-2x_{k}\bar{x}+\bar{x}^{2}\bigr)+P_{k} \overline{\boldsymbol{x}^{2} }+\overline{P}_{k+1} \overline{\boldsymbol{x}^{2}} \\ &{} +\overline{P}_{k+1}\bigl(-2x_{k+1}\bar{x}+ \bar{x}^{2}\bigr)-2\sum_{i=k+2}^{n}\overline{P}_{i}x_{i}x_{i-1}+2\sum _{i=k+2}^{n}\overline{P}_{i}x_{i-1}^{2}. \end{aligned}$$

Applying (3.6) on ***p*** and $\boldsymbol{x}^{2}= ( x_{1}^{2},\ldots,x_{n}^{2} ) $, we obtain

$$\sum_{i=1}^{k-1}P_{i} \bigl( x_{i}^{2}-x_{i+1}^{2} \bigr) +P_{k}\bigl(x_{k} ^{2}-\overline{ \boldsymbol{x}^{2}}\bigr)+\overline{P}_{k+1} \bigl(x_{k+1}^{2}-\overline{\boldsymbol{x}^{2}} \bigr)+\sum_{i=k+2}^{n}\overline {P}_{i} \bigl( x_{i}^{2}-x_{i-1}^{2} \bigr) =0, $$

hence

$$\begin{aligned} I_{k} ={}& 2\sum_{i=1}^{k-1}P_{i}x_{i+1} ( x_{i+1}-x_{i} ) +2\sum_{i=k+2}^{n} \overline{P}_{i}x_{i-1} ( x_{i-1}-x_{i} ) +P_{k}\overline{\boldsymbol{x}^{2}}+\overline{P}_{k+1} \overline {\boldsymbol{x}^{2}} \\ &{} +P_{k}\bigl(-2x_{k}\bar{x}+\bar{x}^{2}\bigr)+ \overline {P}_{k+1}\bigl(-2x_{k+1}\bar{x}+ \bar{x}^{2}\bigr) \\ ={}&2\sum_{i=1}^{k-1}P_{i}x_{i+1} ( x_{i+1}-x_{i} ) +2\sum_{i=k+2}^{n} \overline{P}_{i}x_{i-1} ( x_{i-1}-x_{i} ) +\overline{\boldsymbol{x}^{2}} \\ &{} +P_{k}\bigl(-2x_{k}\bar{x}+\bar{x}^{2}\bigr)+ \overline {P}_{k+1}\bigl(-2x_{k+1}\bar{x}+ \bar{x}^{2}\bigr). \end{aligned}$$

Taking into account that ***x*** is increasing and

$$\begin{aligned} &P_{i}, \overline{P}_{i} \geq0,\quad i=1,2,\ldots,n, \\ &x_{i} \leq\bar{x},\quad i=1,2,\ldots,k, \\ &\bar{x} \leq x_{i},\quad i=k+1,\ldots,n, \end{aligned}$$

we obtain

$$\begin{aligned} I_{k} \leq{}&2\bar{x}\sum_{i=1}^{k-1}P_{i} ( x_{i+1}-x_{i} ) +2\bar{x}\sum_{i=k+2}^{n} \overline{P}_{i} ( x_{i-1}-x_{i} ) +\overline{ \boldsymbol{x}^{2}} \\ &{} -2P_{k}x_{k}\bar{x}-2\overline{P}_{k+1}x_{k+1} \bar{x}+\bar{x}^{2}. \end{aligned}$$

Applying again (3.6) on ***p*** and ***x***, we get

$$\sum_{i=1}^{k-1}P_{i} ( x_{i+1}-x_{i} ) +\sum_{i=k+2}^{n}\overline{P}_{i} ( x_{i-1}-x_{i} ) =P_{k} ( x_{k}-\bar {x} ) +\overline{P}_{k+1} ( x_{k+1}-\bar{x} ), $$

hence

$$\begin{aligned} I_{k} & \leq2\bar{x} \bigl[ P_{k} ( x_{k}- \bar{x} ) +\overline {P}_{k+1} ( x_{k+1}-\bar{x} ) \bigr] + \overline {\boldsymbol{x}^{2}}-2P_{k}x_{k} \bar{x}-2\overline{P}_{k+1}x_{k+1}\bar{x}+\bar {x}^{2} \\ & =-2P_{k}\bar{x}^{2}-2\overline{P}_{k+1} \bar{x}^{2}+\bar{x}^{2}+\overline{ \boldsymbol{x}^{2}}=-2\bar{x}^{2}+\bar{x}^{2}+ \overline {\boldsymbol{x}^{2}}=\overline{\boldsymbol{x}^{2}}- \bar{x}^{2}, \end{aligned}$$

or written differently

$$I_{k}\leq\sum_{i=1}^{n}p_{i}x_{i}^{2}- \bar{x}^{2}=\sum_{i=1}^{n}p_{i}(x_{i}- \bar{x})^{2}. $$

 □

We have just proven that the Jensen–Steffensen inequality for strongly convex functions behaves differently than the Jensen inequality for strongly convex functions: applying the same proof techniques, we end up with two different bounds, and surprisingly the indirect proof gives the better one.

## Integral version

The integral version of the Jensen–Steffensen inequality for convex functions was proved by Boas in 1970 [2].

### Theorem 5

*Let* $x: [ \alpha,\beta ] \rightarrow ( a,b ) $ *be a continuous and monotonic function*, *where* $-\infty<\alpha<\beta <+\infty$ *and* $-\infty\leq a< b\leq+\infty$, *and let* $f: ( a,b ) \rightarrow \mathbb{R} $ *be a convex function*. *If* $\lambda: [ \alpha,\beta ] \rightarrow \mathbb{R} $ *is either continuous or of bounded variation satisfying*

$$\begin{aligned} &\bigl( \forall t\in [ \alpha,\beta ] \bigr) \quad \lambda ( \alpha ) \leq\lambda ( t ) \leq\lambda ( \beta ), \\ &\lambda ( \beta ) -\lambda ( \alpha ) >0, \end{aligned}$$

*then*

$$f \biggl( \frac{\int_{\alpha}^{\beta}x ( t ) \,\mathrm{d}\lambda ( t ) }{\int_{\alpha}^{\beta}\,\mathrm{d}\lambda ( t ) } \biggr) \leq\frac{\int_{\alpha}^{\beta}f ( x ( t ) ) \,\mathrm{d}\lambda ( t ) }{\int_{\alpha}^{\beta} \,\mathrm{d}\lambda ( t ) }. $$

Since the indirect proof as in Theorem 2 produced a better bound, we will use the same technique to prove the integral version of the Jensen–Steffensen inequality for strongly convex functions.

### Theorem 6

*Let* $x: [ \alpha,\beta ] \rightarrow ( a,b ) $ *be a continuous and monotonic function*, *where* $-\infty<\alpha<\beta <+\infty$ *and* $-\infty\leq a< b\leq+\infty$, *and let* $f: ( a,b ) \rightarrow \mathbb{R} $ *be a strongly convex function with modulus* *c*. *If* $\lambda: [ \alpha ,\beta ] \rightarrow \mathbb{R} $ *is either continuous or of bounded variation satisfying*

$$\begin{aligned} &\bigl( \forall t\in [ \alpha,\beta ] \bigr)\quad \lambda ( \alpha ) \leq\lambda ( t ) \leq\lambda ( \beta ), \\ &\lambda ( \beta ) -\lambda ( \alpha ) >0, \end{aligned}$$

*then*

$$f ( \mu ) \leq\frac{\int_{\alpha}^{\beta}f ( x ( t ) ) \,\mathrm{d}\lambda ( t ) }{\int_{\alpha }^{\beta }\,\mathrm{d}\lambda ( t ) }-c\frac{\int_{\alpha}^{\beta } ( x ( t ) -\mu ) ^{2}\,\mathrm{d}\lambda ( t ) }{\int_{\alpha}^{\beta}\,\mathrm{d}\lambda ( t ) }, $$

*where*

$$\mu=\frac{\int_{\alpha}^{\beta}x ( t ) \,\mathrm {d}\lambda ( t ) }{\int_{\alpha}^{\beta}\,\mathrm{d}\lambda ( t ) }. $$

### Proof

Using the convex representation $g=f-c ( \cdot ) ^{2}$ as in Theorem 1 and applying the integral Jensen–Steffensen inequality for convex functions, we obtain

$$g ( \mu ) =g \biggl( \frac{\int_{\alpha}^{\beta}x ( t ) \,\mathrm{d}\lambda ( t ) }{\int_{\alpha}^{\beta}\,\mathrm {d}\lambda ( t ) } \biggr) \leq\frac{\int_{\alpha}^{\beta }g ( x ( t ) ) \,\mathrm{d}\lambda ( t ) }{\int _{\alpha }^{\beta}\,\mathrm{d}\lambda ( t ) }. $$

Going back to *f* we get

$$f ( \mu ) -c\mu^{2}\leq\frac{\int_{\alpha}^{\beta } ( f ( x ( t ) ) -cx ( t ) ^{2} ) \,\mathrm {d}\lambda ( t ) }{\int_{\alpha}^{\beta}\,\mathrm{d}\lambda ( t ) }=\frac{\int_{\alpha}^{\beta}f ( x ( t ) ) \,\mathrm{d}\lambda ( t ) }{\int_{\alpha}^{\beta}\,\mathrm {d}\lambda ( t ) }-c \frac{\int_{\alpha}^{\beta}x ( t ) ^{2}\,\mathrm{d}\lambda ( t ) }{\int_{\alpha}^{\beta }\,\mathrm{d}\lambda ( t ) },$$

or written differently

$$\begin{aligned} f ( \mu ) & \leq\frac{\int_{\alpha}^{\beta}f ( x ( t ) ) \,\mathrm{d}\lambda ( t ) }{\int_{\alpha }^{\beta }\,\mathrm{d}\lambda ( t ) }-c\frac{\int_{\alpha}^{\beta }x ( t ) ^{2}\,\mathrm{d}\lambda ( t ) }{\int_{\alpha }^{\beta }\,\mathrm{d}\lambda ( t ) }+c\mu^{2} \\ & =\frac{\int_{\alpha}^{\beta}f ( x ( t ) ) \,\mathrm{d}\lambda ( t ) }{\int_{\alpha}^{\beta}\,\mathrm {d}\lambda ( t ) }-c \biggl[ \frac{\int_{\alpha}^{\beta }x ( t ) ^{2}\,\mathrm{d}\lambda ( t ) }{\int_{\alpha }^{\beta }\,\mathrm{d}\lambda ( t ) }-\mu^{2} \biggr] \\ & =\frac{\int_{\alpha}^{\beta}f ( x ( t ) ) \,\mathrm{d}\lambda ( t ) }{\int_{\alpha}^{\beta}\,\mathrm {d}\lambda ( t ) }-c \biggl[ \frac{\int_{\alpha}^{\beta }x ( t ) ^{2}\,\mathrm{d}\lambda ( t ) }{\int_{\alpha }^{\beta }\,\mathrm{d}\lambda ( t ) }-2\mu^{2}+ \mu^{2} \biggr] \\ & =\frac{\int_{\alpha}^{\beta}f ( x ( t ) ) \,\mathrm{d}\lambda ( t ) }{\int_{\alpha}^{\beta}\,\mathrm {d}\lambda ( t ) }-c \biggl[ \frac{\int_{\alpha}^{\beta }x ( t ) ^{2}\,\mathrm{d}\lambda ( t ) }{\int_{\alpha }^{\beta }\,\mathrm{d}\lambda ( t ) }-2\mu\frac{\int_{\alpha }^{\beta}x ( t ) \,\mathrm{d}\lambda ( t ) }{\int_{\alpha}^{\beta }\,\mathrm{d}\lambda ( t ) }+ \mu^{2}\frac{\int_{\alpha }^{\beta }\,\mathrm{d}\lambda ( t ) }{\int_{\alpha}^{\beta}\,\mathrm {d}\lambda ( t ) } \biggr] \\ & =\frac{\int_{\alpha}^{\beta}f ( x ( t ) ) \,\mathrm{d}\lambda ( t ) }{\int_{\alpha}^{\beta}\,\mathrm {d}\lambda ( t ) }-c\frac{\int_{\alpha}^{\beta} ( x ( t ) -\mu ) ^{2}\,\mathrm{d}\lambda ( t ) }{\int _{\alpha }^{\beta}\,\mathrm{d}\lambda ( t ) }. \end{aligned}$$

 □
